# System‐Level Analysis of Japan's Pediatric–Perinatal Disaster Liaison Operations During the 2024 Noto Earthquake

**DOI:** 10.1111/ped.70488

**Published:** 2026-07-13

**Authors:** Kyosuke Kagami, Kazunori Imai, Yasuhisa Ueno, Hiroyuki Kitano, Shinya Hirabuki, Hiromasa Sasaki, Hitoshi Sato, Yusuke Mitani, Hidenori Iwasaki, Taizo Wada, Hiroshi Fujiwara, Nobuya Unno

**Affiliations:** ^1^ Department of Obstetrics and Gynecology, Graduate School of Medical Sciences Kanazawa University Kanazawa Ishikawa Japan; ^2^ Department of Emergency and Critical Care, Graduate School of Medical Sciences Nagoya City University Nagoya Japan; ^3^ Department of Neonatology, Medical Center for Maternal and Child Health Ishikawa Prefectural Central Hospital Kanazawa Ishikawa Japan; ^4^ Department of Obstetrics and Gynecology Ishikawa Prefectural Central Hospital Kanazawa Ishikawa Japan; ^5^ Department of Pediatrics, Faculty of Medicine Kanazawa Medical University Kanazawa Ishikawa Japan; ^6^ Department of Pediatrics, Graduate School of Medicine, Institute of Medical, Pharmaceutical and Health Sciences Kanazawa University Kanazawa Ishikawa Japan; ^7^ Ochi Yume Clinic Nagoya Japan; ^8^ School of Veterinary Medicine Azabu University Sagamihara Japan; ^9^ Center for Perinatal Medicine JCHO Sagamino Hospital Kanazawa Japan

**Keywords:** disaster medicine, earthquake, Japan, maternal health services, pediatric care

## Abstract

**Background:**

Children, neonates, and pregnant women are particularly vulnerable during disasters. Fragmentation between specialized pediatric–perinatal systems and general disaster response frameworks can hinder coordinated care. Following lessons from the 2011 Great East Japan Earthquake, Japan established the Disaster Liaison for Pediatric and Perinatal Medicine (DLPPM) to embed specialists within disaster command structures. However, large‐scale activation under prolonged infrastructure disruption has not been systematically evaluated.

**Methods:**

We conducted a structured retrospective descriptive analysis of DLPPM operational records during the first month after the 2024 Noto Peninsula Earthquake. Activities were reviewed across five pre‐specified domains to examine how the liaison framework functioned during the acute and subacute phases.

**Results:**

DLPPM was integrated into the prefectural disaster headquarters and consolidated maternal–child health information, enabling centralized identification of 83 pregnant women, estimated to represent most pregnant women in the severely affected region. Twenty‐one obstetric transfers were coordinated. Pediatric transfers and evacuation of medically dependent children were facilitated through established networks. During the subacute phase, DLPPM initiated maternal–child support measures, including a “Children's Conference” and a support website. These findings suggest that DLPPM functioned as a centralized coordination hub linking specialized clinical networks with disaster governance, although real‐time identification of vulnerable families in shelters remained limited.

**Conclusions:**

Embedding pediatric and perinatal specialists within disaster headquarters can support structured medical coordination for vulnerable populations. Earlier and more systematic integration with public health and welfare systems is essential to extend this hub function beyond hospital‐centered care.

## 1 Introduction

Children, neonates, and pregnant women are disproportionately vulnerable during disasters because of their physiological characteristics and reliance on highly specialized and continuous care systems [1]. Pediatric patients have unique airway, metabolic, and pharmacologic considerations that complicate emergency management, and neonates in intensive care depend on uninterrupted electricity, thermal regulation, respiratory support, and coordinated transport capacity [2]. Pregnant and postpartum women face additional risks when prenatal care continuity is disrupted and living conditions deteriorate, including physical and psychosocial vulnerability [3]. Protecting these populations therefore requires systems capable of sustaining specialized pediatric and perinatal care under unstable conditions.

International disaster experiences have demonstrated that adverse outcomes in maternal and child populations are often attributable not only to resource scarcity but also to fragmentation across systems. Even within hospital settings, evacuation and interfacility transfers may falter when shared strategies, communication pathways, and command relationships are not clearly defined [4]. These observations indicate that specialized pediatric–perinatal care networks do not automatically integrate into general disaster response frameworks and may require an explicit bridging mechanism to connect clinical expertise with administrative decision‐making and operational logistics.

In Japan, the Great East Japan Earthquake in 2011 revealed significant challenges in coordinating pediatric and perinatal healthcare within the broader disaster medical response framework. Reports described fragmented information sharing, limited centralized data aggregation, and difficulties aligning specialized care needs with disaster response operations [5]. These experiences highlighted the need for a clearer interface between pediatric–perinatal expertise and disaster coordination structures.

In response, Japan established the Disaster Liaison for Pediatric and Perinatal Medicine (DLPPM) system in 2016. DLPPM embeds pediatric and perinatal specialists within disaster response headquarters, with core functions including centralized information consolidation, medical coordination, and maternal–child health support. Formal activity guidelines were issued in 2019, and the system was further institutionalized in 2022, when prefectures began officially appointing liaison specialists at disaster coordination headquarters [6]. Since its establishment, DLPPM has been activated in multiple disasters across Japan, gradually refining its coordination framework through accumulated field experience (Table 1). Early deployments primarily focused on medical consultation and patient transfer support, whereas later activations increasingly incorporated maternal–child health coordination, community‐based surveillance, and collaboration with public health and welfare sectors. This progressive expansion suggests that the liaison role has shifted from a predominantly clinical support function toward a broader maternal–child protection framework integrating medical and public health domains. However, despite this gradual functional evolution, large‐scale prefectural implementation under conditions of prolonged infrastructure disruption had not been systematically described or evaluated. The 2024 Noto Peninsula Earthquake (moment magnitude [Mw] 7.5) provided the first full‐scale prefectural activation of the DLPPM system under conditions of geographic isolation and sustained infrastructure disruption. The northern Noto region, characterized by depopulation, advanced aging, and limited maternal and pediatric healthcare capacity, was particularly vulnerable. Obstetric services were scarce, and pediatric inpatient care was restricted, making regional coordination essential following widespread infrastructure damage.

**TABLE 1:** Evolution of the DLPPM system through successive disasters in Japan.

Disaster/milestone	System development	Information and coordination	Medical support	Public health and welfare
2016 – DLPPM established	Concept of pediatric–perinatal disaster liaison introduced	Initial communication framework	Planning for specialist‐led coordination	Recognition of maternal–child vulnerability
2018 Kumamoto Earthquake	First operational experience	EMIS integration, facility mapping	Physician dispatch, transfer coordination	Maternal supply distribution
2018 Osaka Northern Earthquake	Expansion of coordination model	PEACE integration	PICU transfer coordination support	
2018 Western Japan Floods	Strengthening inter‐regional networks	EMIS, PEACE integration	Home care support, pediatric support	Shelter‐based support
2018 Hokkaido Eastern Iburi Earthquake	Expansion of perinatal–pediatric coordination	Perinatal emergency coordination, SDF transport, power supply disruptions	Transfer coordination, Home‐based medical support	Infant formula supply, vaccination coordination
2019 Typhoon Faxai	Consolidation of cross‐sector collaboration	Pediatrics and perinatal coordination	Perinatal facility coordination	Allergy support, nutrition guidance, mental support
2024 Noto Peninsula Earthquake	First large‐scale full implementation	EMIS, PEACE, J‐SPEED, D24H integration; real‐time communication via messaging platforms	Transfer coordination, evacuation of medically dependent children	Children's Conference, maternal–child health support website, infant formula supply

Abbreviations: D24H, Disaster Digital Information System for Health and Well‐being; DLPPM, Disaster Liaison for Pediatric and Perinatal Medicine; EMIS, Emergency Medical Information System; J‐SPEED, Japan Surveillance in Post Extreme Emergencies and Disasters; PEACE, Perinatal Emergency Activity System for Collaboration and Exchange; PICU, Pediatric Intensive Care Unit; SDF, Japan Self‐Defense Forces.

This event offers a crucial opportunity to examine how a pediatric–perinatal liaison hub functioned under real‐world disaster conditions, to identify operational achievements and enabling structural factors, and to clarify persistent gaps at the interface between medical coordination and community‐based maternal–child protection. The present study documents DLPPM activities during the acute and subacute phases of the Noto Peninsula Earthquake and provides a structured retrospective system‐level evaluation to inform future pediatric and perinatal disaster preparedness strategies.

## 2 Methods

### 2.1 Study Design and Setting

This study was a structured retrospective descriptive system‐level evaluation of DLPPM activities conducted during the first month following the 2024 Noto Peninsula Earthquake. Using operational records from the disaster response, we examined how the liaison framework functioned within the prefectural disaster headquarters, focusing on coordination processes, information flows, and operational decision‐making across acute and subacute phases. This study was intended to characterize operational functions and system‐level challenges of DLPPM rather than to test intervention effectiveness.

### 2.2 Data Sources and Collection

Information on DLPPM activities was obtained primarily from chronological operational logs during the disaster response by liaison team members at the prefectural disaster headquarters. These records documented coordination episodes, communication summaries, transfer arrangements, and support activities. When clarification of chronology or operational details was required, supplementary confirmation was obtained from DLPPM liaison officers directly involved in the relevant coordination. Supplementary confirmation was used to clarify recorded events rather than to reconstruct unrecorded activities whenever possible. Information regarding pregnant women was collected from municipal maternal–child health records and regional obstetric facilities. These data were integrated into a centralized registry maintained by DLPPM. Patient‐related information was limited to cases in which medical transfers or care coordination were directly supported by DLPPM. All data were anonymized prior to analysis, and no personally identifiable information was included.

### 2.3 Ethical Considerations

This study was conducted in accordance with the Declaration of Helsinki and the Ethical Guidelines for Medical and Health Research Involving Human Subjects in Japan. Because the study was based on retrospective analysis of anonymized operational records and did not involve direct patient intervention, informed consent was waived using an opt‐out procedure disclosed on institutional websites. The study protocol was approved by the Medical Ethics Committee of Kanazawa University (Approval No. 714721‐1).

### 2.4 Data Analysis

This study employed a structured retrospective system‐level evaluation that drew on basic principles of the World Health Organization (WHO) After Action Review (AAR) for structuring post‐event analysis [7]. These principles were used to inform the analytical structure rather than to conduct a formal WHO AAR. Operational records were reviewed chronologically to reconstruct response activities and identify key operational functions performed by the DLPPM. Based on the core functions of the DLPPM described in its operational guidelines and previous reports, five evaluation domains were specified before detailed data extraction: (1) information integration, (2) medical coordination and transfer support, (3) mobilization of professional and community networks, (4) transition from acute response to sustained maternal–child support, and (5) persistent gaps in local integration and community coordination. Operational activities and observations were then mapped to the most relevant domain primarily by the first and second authors, with input from the third author when needed. Ambiguous classifications were resolved through discussion. The analysis focused on documenting observed activities, identifying strengths and operational challenges, and extracting lessons for future disaster preparedness and response. A summary matrix linking representative activities, indicators, achievements, and identified challenges across the five domains was subsequently developed to enhance transparency of the analytical framework.

### 2.5 Use of Artificial Intelligence Tools

Artificial intelligence (AI)–based language tools were used solely for language editing and translation support during manuscript preparation. Specifically, ChatGPT (OpenAI) and DeepL Translator (DeepL SE) were utilized to assist with English grammar correction, phrasing refinement, and translation. These tools were not used for data analysis, data interpretation, or generation of scientific content. All study design, data handling, analysis, interpretation, and final manuscript content were performed and verified by the authors.

## 3 Results

### 3.1 Disaster Context and Initial Activation of DLPPM Within the Prefectural Headquarters

**FIGURE 1:**
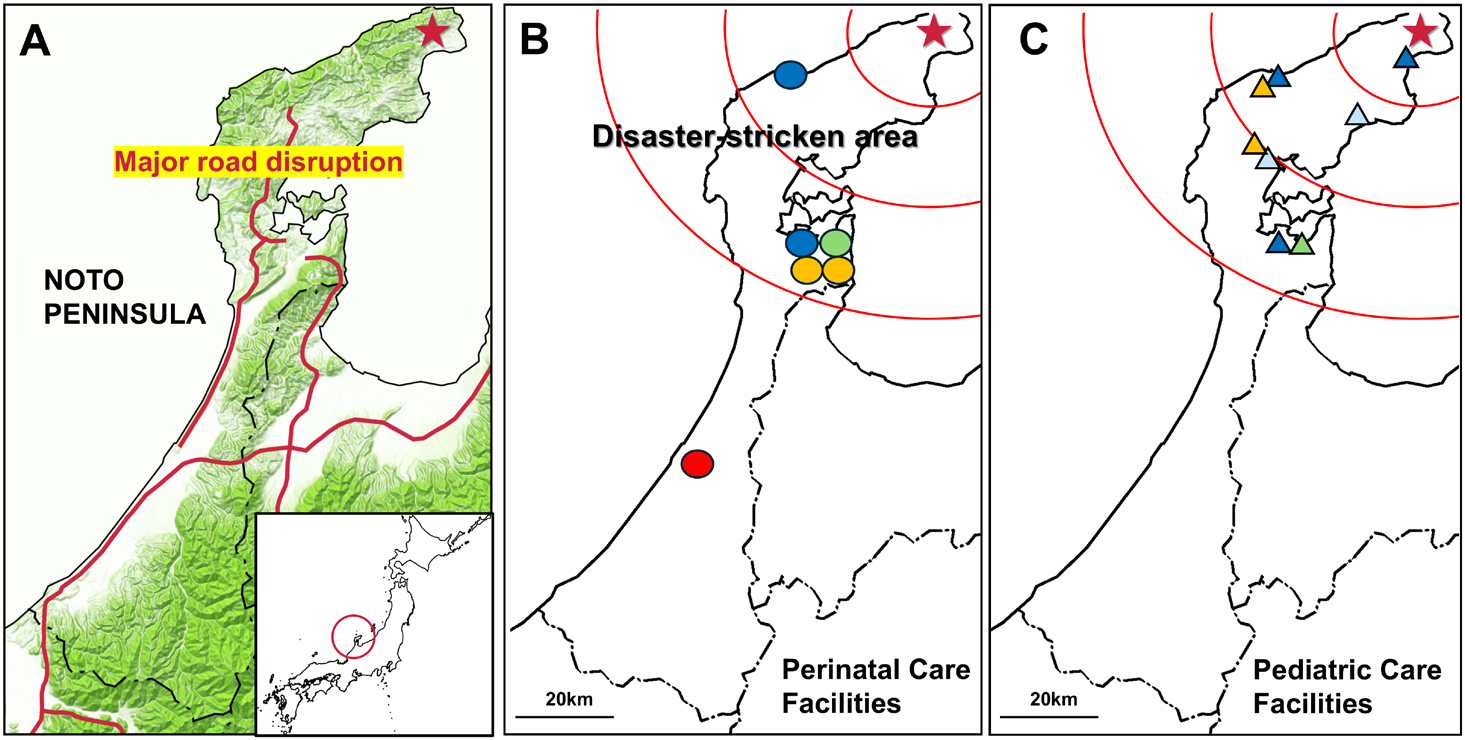
Maps of the Noto Peninsula region affected by the 2024 earthquake. (A) Topographic map of the Noto Peninsula indicating road closures (yellow highlight) following the earthquake. The red star marks the epicenter, and red lines indicate major arterial roads, including expressways. (B) Distribution of perinatal care facilities. Blue circles represent public general hospitals, green circles represent private general hospitals, and yellow circles indicate obstetric clinics. The red circle indicates the Comprehensive Perinatal Care Center at the Ishikawa Prefectural Disaster Base Hospital in Kanazawa City. (C) Distribution of pediatric care facilities. Dark blue triangles represent public general hospitals with inpatient pediatric services. Light blue triangles represent public general hospitals providing outpatient pediatric services only. Yellow triangles indicate pediatric clinics, and green triangles represent private general hospitals.

The Noto Peninsula Earthquake (Mw 7.5) struck northern Ishikawa Prefecture at 4:10 p.m. on January 1, 2024, affecting a sparsely populated region characterized by advanced aging and depopulation. The earthquake caused widespread infrastructure damage, including road collapse, tsunami waves, ground deformation, and prolonged disruption of essential utilities such as water and gas, resulting in geographic isolation of multiple coastal and mountainous communities (Figure 1A).

Even prior to the disaster, maternal and pediatric healthcare capacity in the northern Noto region was limited. Obstetric services were scarce, with no private obstetric clinics and only one general hospital providing a small number of deliveries per month. Most pregnant women planned delivery in Nanao City, a regional hub with two general hospitals and two obstetric clinics (Figures 1B and S1). Pediatric care capacity was similarly constrained, with inpatient pediatric services available only at hospitals in Suzu and Wajima, and critically ill children routinely referred to tertiary centers in Kanazawa (Figure 1C).

Within approximately 1 h of the 4:10 p.m. earthquake, DLPPM was activated at the Ishikawa Prefectural Government Office at 5:15 p.m. Pediatricians, neonatologists, and obstetricians were integrated into the prefectural disaster response headquarters to coordinate pediatric and perinatal care. A group messaging platform was used to facilitate communication among specialists and relevant stakeholders. During the first 2 months following the disaster, 12 physicians (three pediatricians, four neonatologists, and five obstetricians) participated in DLPPM activities. A chronological overview of the major DLPPM activities during the disaster response is presented in Table 2. For system‐level evaluation, these activities were retrospectively organized into five pre‐specified domains reflecting the system's core operational functions. A summary of the major activities, representative indicators, achievements, and identified challenges across these domains is presented in Table 3.

**TABLE 2:** Chronology of major DLPPM activities following the 2024 Noto Peninsula Earthquake.

Post‐disaster day	Major activity	Operational significance
Day 1	DLPPM activated within the prefectural disaster headquarters	Integration of pediatric, neonatal, and obstetric specialists into disaster command
Days 1–17	Coordination of 21 obstetric‐related transfers (including 9 helicopter evacuations)	Maintenance of safe access to perinatal care
Days 1–7	Coordination of seven pediatric transfers and evacuation/transfer of four medically dependent children	
Days 3–14	Coordination of maternal–child relief supplies, including infant formula, medicines, and clean water	Supported continuity of infant feeding and essential maternal–child health needs in affected communities
Day 6–140	Identification, centralized registration of 83 pregnant women and follow‐up	Continuous tracking of locations, evacuation destinations, planned delivery facilities, and healthcare status
Days 6–60	Continuous collection and integration of information from municipal maternal–child health services, regional healthcare facilities, D24H, DHEAT, DMAT, and other field networks	Supported situational awareness and informed operational decision‐making for maternal–child health coordination throughout the disaster response
Days 6–90	Establishment and operation of the Children's Conference	Created a multidisciplinary coordination platform linking healthcare, welfare, education, and administrative sectors, supporting child health and welfare needs through the subacute phase
Days 18–20	Implementation of measures for early identification of and response to sexual violence, including emergency contraceptive pill distribution	Enhanced protection of women's health and safety during the subacute phase
Day 36 onward	Launch of the maternal–child health support website	Improved access to health information and consultation services

Abbreviations: D24H, Disaster/Digital Information System for Health and Well‐being; DHEAT, Disaster Health Emergency Assistance Team; DLPPM, Disaster Liaison for Pediatrics and Perinatal Medicine; DMAT, Disaster Medical Assistance Teams.

**TABLE 3:** Structured evaluation of DLPPM operations during the 2024 Noto Peninsula Earthquake according to five pre‐specified system‐level domains.

Evaluation domain	Major activities	Representative indicators	Major achievements	Identified challenges
1. Information Integration	Integrated maternal–child health records from municipalities and clinical information from healthcare facilities into a centralized database managed within the prefectural disaster headquarters.	83 pregnant women identified and registered Centralized database continuously updated	Enabled comprehensive regional situational awareness by maintaining a centralized database that tracked the whereabouts, evacuation destinations, healthcare status, and delivery arrangements of pregnant women throughout the disaster response.	The centralized registry approach was feasible in a rural region with a relatively small pregnant population; large‐scale urban disasters would require alternative scalable systems for maternal–child situational awareness and population tracking.
2. Medical Coordination and Transfer Support	Coordinated obstetric transport, evacuation planning for women ≥ 36 weeks' gestation, and pediatric referral support through disaster headquarters.	21 obstetric transport cases coordinated 9 helicopter evacuations 7 pediatric transfer cases	Enabled timely transfer of vulnerable pregnant women and children despite severe transportation disruption; no out‐of‐hospital births were reported.	The transfer strategy relied on preserved healthcare capacity in southern Ishikawa Prefecture; disasters affecting wider geographic areas may require inter‐prefectural or national‐level evacuation and transfer frameworks.
3. Network Mobilization Across Professional and Community Stakeholders	Activated existing pediatric, neonatal, obstetric, welfare, and community networks; collaborated with DMAT, ICNCV, Konokono, municipalities, and Self‐Defense Forces.	Information sharing through a regional pediatric mailing network Four medically dependent children evacuated or transferred	Existing professional and community networks facilitated rapid identification and rescue of medically vulnerable children.	Support was largely limited to technology‐dependent children; broader networks, redundant communication systems, and individualized evacuation planning are needed for more diverse vulnerable populations.
4. Acute‐to‐Subacute Transition Functions	Coordinated breastfeeding support, infant nutrition, supply distribution, mental health support, establishment of the Children's Conference, and launch of a maternal–child support website.	Children's Conference established Maternal–child support website launched	Established a cross‐sector coordination platform that facilitated information sharing, joint decision‐making, and sustained maternal–child support beyond the acute phase.	Formal integration of cross‐sector networks into regional preparedness planning and development of accessible digital maternal–child support systems remain important future priorities.
5. Persistent Gaps in Local Integration and Community Coordination	Attempted to collect information from shelters, public health centers, municipalities, and disaster medical teams regarding vulnerable mothers and children.	Limited information available from shelters and unofficial evacuation sites Delayed establishment of welfare shelters	Identified structural weaknesses in community‐level situational awareness and maternal–child welfare coordination.	Pre‐disaster integration of liaison personnel into local maternal–child health networks and disaster coordination systems remain an important future priority.

Abbreviations: DLPPM, Disaster Liaison for Pediatrics and Perinatal Medicine; DMAT, Disaster Medical Assistance Teams; ICNCV, the Ishikawa Community Network for Children with Ventilators. Konokono refers to the Ishikawa Support Center for Technology‐dependent Children.

### 3.2 Acute‐Phase Information Integration and Obstetric Coordination

**FIGURE 2:**
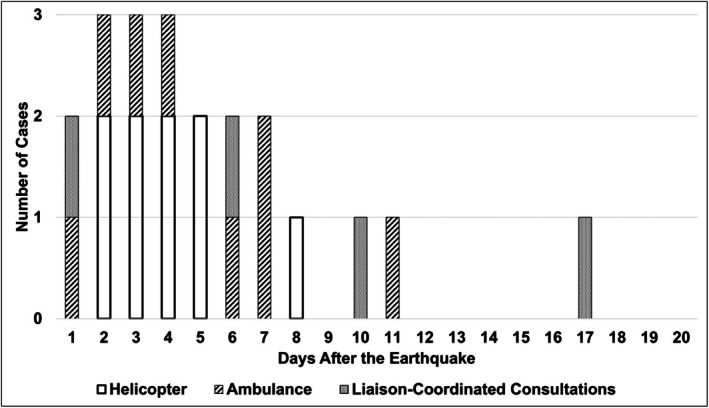
Perinatal Transport Methods by Day Following the Noto Peninsula Earthquake. A bar graph showing daily counts of perinatal transport following the Noto Peninsula Earthquake. “Liaison‐Coordinated Consultation” indicates cases in which DLPPM provided transport coordination or care advice via remote communication.

During the acute phase, DLPPM implemented information integration and medical coordination for pregnant women and obstetric care needs in the affected region. A total of 83 pregnant women were identified, representing nearly all pregnant women in the severely affected northern Noto region. This determination was based on municipal maternal–child health registries and information obtained from regional obstetric facilities. These individuals were registered in a centralized database developed and managed by DLPPM. The registry enabled continuous tracking of pregnant women's locations, evacuation destinations, planned delivery facilities, and healthcare status throughout the disaster response. The database was continuously updated through collaboration with local authorities, healthcare providers, and a pre‐existing perinatal network centered in Nanao City. For women at or beyond 36 weeks of gestation, evacuation outside the disaster area was recommended to ensure safe delivery, and this recommendation was communicated through local healthcare facilities. By January 17, a total of 21 obstetric‐related transport cases had been coordinated, including nine evacuations by helicopter (Figure 2; Table S1). Notably, no out‐of‐hospital births were reported in the affected region during the acute phase.

### 3.3 Network Mobilization for Pediatric and Medically Complex Child Evacuation

DLPPM leveraged professional and community networks to identify children requiring specialized care unavailable in the affected region and to coordinate transfer or evacuation. Within the first 7 days following the earthquake (by January 7), DLPPM coordinated seven pediatric cases, including spinal muscular atrophy type I, trauma, post‐hematopoietic stem cell transplantation management, and metabolic disease, with transfers to tertiary hospitals in Kanazawa. A regional mailing list involving pediatricians in Ishikawa Prefecture and neighboring areas was newly established by the Department of Pediatrics at Kanazawa University, creating a communication pathway through which field information could be relayed to DLPPM. In addition, DLPPM coordinated the evacuation and/or hospital transfer of four children with medical complexity, including technology‐dependent children at risk of life‐threatening deterioration, in collaboration with Disaster Medical Assistance Teams (DMAT), Japan's national deployable disaster medical teams, the Ishikawa Community Network for Children with Ventilators (ICNCV), the Ishikawa Medical Support Center for Technology‐dependent Children (“Konokono”), and the Self‐Defense Forces (Table 4). Information on these children was initially shared through existing community‐based networks and subsequently relayed to DLPPM. One case involved a child receiving home oxygen therapy who became isolated due to road collapse. With land access unavailable, helicopter evacuation was arranged shortly before oxygen depletion.

**TABLE 4:** Evacuation and medical coordination for four medically dependent children during the acute phase of the Noto Peninsula Earthquake.

Case	Medical condition (Age)	Residence area	Timeline after disaster onset			
			Day 1	Day 2	Day 3	Day 4
1	Nocturnal mechanical ventilation (11 years old)	Noto region (North)	Safety unknown	Safety confirmed to be evacuated safely by the oxygen supply company		
2	Nocturnal mechanical ventilation (24 years old)	Noto region (North)	Safety unknown	Risk of isolation due to disrupted lifelines (water, electricity); transported and hospitalized		
3	Congenital heart disease, Home oxygen therapy (11 years old)	Noto region (North)	Safety unknown	Safety unknown	Evacuated and hospitalized with support from welfare staff of “Konokono,” the Ishikawa Support Center for Medically Dependent Children	
4	Congenital heart disease, Home oxygen therapy (2 years old)	Noto region (North)	Safety unknown	Due to house collapse and community isolation, relied on car battery for power supply	Continued isolation, with information received that car battery power would be depleted the next day; attempt to deliver oxygen by land failed due to inaccessible roads	Rescued by helicopter hoist operation and transported to hospital

### 3.4 Transition to Subacute Maternal–Child Health and Welfare Coordination

In addition to medical coordination, DLPPM addressed maternal and child health and welfare needs during the disaster response. Activities included breastfeeding support, infant feeding coordination, and provision of essential supplies such as infant formula, diapers, medicines, and clean water. In parallel, as no pre‐existing cross‐sectoral forum focusing on child health and welfare had been in place, DLPPM proposed the establishment of a cross‐sectoral coordination forum termed the “Children's Conference” on January 6, and the first meeting was convened on January 9. The conference included pediatricians, obstetricians, mental health professionals, representatives from prefectural and municipal administrations, public health authorities, education‐related personnel, and child welfare stakeholders. The “Children's Conference” facilitated child health and welfare support, including health management in group evacuations, arrangements for allergen‐free diets for children with food allergies, and mental health care for displaced students. Prolonged displacement further affected community life. Several schools were repurposed as evacuation shelters, and approximately 400 junior high school students from Wajima, Suzu, and Noto were relocated to external locations between mid‐January and March under supervised group arrangements that included mental health support [8]. The Children's Conference also provided consultative support and coordination for these student relocations, including collaboration with education authorities and mental health professionals to address emerging psychosocial and health‐related needs among displaced students.

Concurrently, DLPPM collaborated with the Cabinet Office and the Ishikawa Prefecture Obstetrics and Gynecology Association to implement support measures aimed at facilitating early identification of and response to potential sexual violence in evacuation settings. One month after the disaster, DLPPM launched a maternal–child health support website (available at https://kanazawa‐obgyn.org/boshi2024/) providing self‐check tools and links to consultation services. The website facilitated access to care, including the connection of a postpartum woman with mastitis to midwifery support.

### 3.5 Persistent Gaps in Community‐Level Situational Awareness

Despite these efforts, real‐time community‐level situational awareness for pregnant women, postpartum mothers, and infants remained limited. Information was collected from municipal staff, health professionals, and disaster medical teams; however, updates from shelters were often incomplete, and detailed information from temporary or unofficial evacuation sites was limited. As a result, it was difficult to accurately identify shortages of infant supplies and to locate vulnerable families.

Local administrative staff were heavily engaged in shelter operations and other emergency tasks, limiting their ability to prioritize routine maternal and child health services. In some areas, health centers were used as evacuation facilities, disrupting infant health checkups and parenting support programs, and the establishment of planned welfare shelters for mothers and children was delayed. Information sharing between DLPPM and local administrative staff and medical support teams in the affected areas was also limited. From the DLPPM perspective, this made it difficult to accurately assess on‐site needs, while from the local side, information collected by DLPPM was not always effectively shared or utilized. Although municipalities and healthcare providers continued support through home visits, telephone outreach, and coordination with obstetric institutions, identifying the locations and needs of affected mothers and children remained challenging.

## 4 Discussion

The present structured retrospective system‐level evaluation of DLPPM activities during the 2024 Noto Peninsula Earthquake demonstrated four principal findings. First, DLPPM functioned as a centralized coordination hub that enabled integration of maternal–child health information and coordination of obstetric and pediatric transfers. Second, a centralized registry facilitated longitudinal tracking of nearly all pregnant women in the severely affected region. Third, existing networks supported evacuation of medically dependent children and continuity of specialized care. Finally, DLPPM activities evolved beyond acute medical coordination to include broader maternal–child health and welfare support through the Children's Conference and maternal–child health support website. At the same time, limitations remained in real‐time community‐level information capture and integration with public health and welfare systems.

Pregnant women, neonates, and children are disproportionately vulnerable during disasters because of their physiological characteristics and dependence on continuous, specialized care [9, 10]. In the Noto region, where baseline obstetric and pediatric capacity was already limited, rapid consolidation of clinical information was essential. DLPPM centrally identified and listed 83 pregnant women, representing nearly all pregnant women in the severely affected northern Noto region, while also prioritizing 21 obstetric transfers and coordinating pediatric evacuations, including those of medically dependent children. These activities suggest that concentrating operational information within a designated liaison hub may facilitate timely operational decisions. While comprehensive identification of 83 pregnant women was achievable in the sparsely populated Noto region, replication of this registry‐based approach in densely populated metropolitan disasters would likely require automated data integration and alternative surveillance strategies. In Japan, disaster response resources, including DMAT and inter‐facility transport coordination, are structurally concentrated at prefectural government headquarters, allowing centralized information aggregation to translate efficiently into coordinated action [11]. This institutional configuration likely enhanced the capacity of the liaison hub to function effectively during the acute phase. This hub function was supported not only by formal designation but also by underlying preparedness maturity, including prior training exercises and accumulated field experience since its establishment.

Transfer coordination appears to have been facilitated by multilayered professional and administrative networks developed during peacetime. The successful evacuation of technology‐dependent children was facilitated by existing medical and community support networks. However, the response also highlighted the need to extend preparedness efforts beyond this population. Future planning should strengthen individualized evacuation plans, redundant communication pathways, and community‐based support systems to identify and support children and individuals with a broader range of disabilities and healthcare needs. Collaboration among tertiary centers, regional hospitals, community‐based organizations such as Konokono and ICNCV, and cross‐institutional communication platforms enabled rapid bridging between fragmented information sources and transport decision‐making. Importantly, transfer coordination also depended on the availability of functioning referral centers in southern Ishikawa Prefecture. Because healthcare capacity in the southern part of the prefecture remained intact, patients could be transferred through established regional referral pathways. If the disaster had affected a broader geographic area including these receiving facilities, substantially broader inter‐prefectural coordination mechanisms would likely have been required. These observations suggest that the effectiveness of the liaison role depends on established networks, communication pathways, and referral capacity. As conceptualized by Ito et al., the DLPPM is expected to function as a regional representative within the disaster medical coordination headquarters, bringing localized clinical and community knowledge into centralized decision‐making structures [6]. The Noto experience supports this framework, demonstrating that when a liaison possessing region‐specific information is positioned within the prefectural coordination body, operational decisions can be aligned more effectively with local realities.

Importantly, the liaison mechanism also enabled early transition from acute life‐saving coordination to subacute maternal–child health support. The establishment of the “Children's Conference” and the maternal–child health support website illustrates that intervention during the ultra‐acute phase can anticipate emerging subacute needs. The Children's Conference facilitated coordination across healthcare, welfare, education, and administrative sectors, whereas the website improved access to maternal–child health information and consultation services. This capacity to foresee and prepare for the shift from emergency stabilization to continuity‐of‐care challenges appears to be a key operational strength of the hub model.

However, the Noto response also revealed persistent structural limitations. Real‐time identification of vulnerable mothers and children in community and shelter settings remained difficult, consistent with previous reports highlighting the invisibility of socially vulnerable populations during disasters [12]. Although reporting systems such as the Japan Surveillance in Post Extreme Emergencies and Disasters (J‐SPEED) were utilized to collect health‐related data from evacuation shelters [13], real‐time linkage between these reporting mechanisms and the liaison hub was limited, and perinatal‐specific needs were not systematically captured. While J‐SPEED includes fields relevant to pediatric and perinatal populations, data were not consistently updated, resulting in limited availability of current information and difficulty in identifying evolving maternal–child health needs. Beyond structural limitations of the reporting system, effective data entry also depends on the awareness of personnel responsible for documentation; without adequate recognition of pediatric and perinatal vulnerabilities and the importance of information sharing, proactive reporting may not occur. While hospital‐based coordination was facilitated through the liaison hub, community‐level surveillance and welfare coordination were not equivalently structured. This gap was one contributing factor that limited comprehensive situational awareness beyond the clinical domain. Strengthening both the technical interoperability of reporting systems and training to enhance awareness of maternal–child vulnerabilities may improve real‐time visibility and cross‐sectoral responsiveness in future disasters.

The establishment of DLPPM itself was informed by lessons from the Great East Japan Earthquake, during which coordination challenges in pediatric and perinatal care were widely reported [5]. The Noto experience suggests that embedding a liaison hub within disaster command structures can effectively bridge this gap in the medical domain, as anticipated in the conceptual framework of DLPPM [6]. Furthermore, the response indicates that the liaison model may extend beyond medical transfer coordination toward broader maternal–child protection, representing a potential paradigm shift in the perceived role of pediatric–perinatal disaster systems.

To realize this expanded role, several conditions are necessary. First, disaster preparedness training and exercises must explicitly incorporate liaison functions and cross‐sectoral coordination. Second, multilayered networks—including medical institutions, community organizations, and municipal public health authorities—should be maintained and strengthened during peacetime. Third, predefined communication channels and interoperable reporting strategies should be established before disasters occur. Particularly for effective integration with welfare systems, strategies for real‐time local information capture must be developed to ensure that vulnerable families outside hospital settings are rapidly identified as emphasized in international maternal–child emergency planning frameworks [14].

This study has limitations. It is based on retrospective operational records without a comparison group, and quantitative outcome measures were not systematically evaluated. Operational records may incompletely capture all field activities and informal coordination processes, particularly those not documented in real time. The findings reflect experience in a depopulated rural region and may not directly generalize to urban environments. Nonetheless, this first large‐scale activation of DLPPM demonstrates that a pediatric–perinatal liaison hub can facilitate structured medical coordination and may serve as a foundation for more comprehensive maternal–child disaster protection systems.

## 5 Conclusion

In this structured retrospective system‐level evaluation of DLPPM activities during the 2024 Noto Peninsula Earthquake, the liaison system functioned as a centralized coordination hub linking disaster command structures with specialized pediatric and perinatal care. The evaluation demonstrated its contribution to information integration, medical transfer coordination, mobilization of professional networks, and transition to broader maternal–child health support. At the same time, persistent challenges in real‐time community‐level information capture and cross‐sectoral integration were identified. These findings suggest that strengthening interoperability among medical, public health, and welfare systems will be essential to enhance maternal–child protection during future disasters.

## Author Contributions

K.K., K.I., and Y.U. collected and analyzed the data. K.K. drafted the manuscript. K.I. supervised the study and contributed to its conceptual design and critical revision. K.K., Y.U., H.K., S.H., H.Sa., H.So., Y.M., and H.I. participated in disaster coordination activities as members of the DLPPM. K.I. and N.U. supervised the DLPPM activities. T.W. and H.F. supported implementation of DLPPM operations. All authors reviewed and approved the final manuscript.

## Conflicts of Interest

The authors declare no conflicts of interest.

## Supporting information


**Figure S1:** Administrative map of Ishikawa Prefecture showing municipal boundaries relevant to the 2024 Noto Peninsula Earthquake.
**Table S1:** Perinatal transport cases coordinated during the acute phase of the 2024 Noto Peninsula Earthquake.

## Data Availability

The data that support the findings of this study are available on request from the corresponding author. The data are not publicly available due to privacy or ethical restrictions.
