# Supplementary material for: System‐Level Analysis of Japan's Pediatric–Perinatal Disaster Liaison Operations During the 2024 Noto Earthquake
Source: Pediatr Int. 2026 Jul 13;68(1):e70488. doi: 10.1111/ped.70488 (PMC13358397; doi:10.1111/ped.70488)
Supplement: Supplementary file 1 — Figure S1: Administrative map of Ishikawa Prefecture showing municipal boundaries relevant to the 2024 Noto Peninsula Earthquake. Table S1: Perinatal transport cases coordinated during the acute phase of the 2024 Noto Peninsula Earthquake. [file PED-68-e70488-s001.docx]

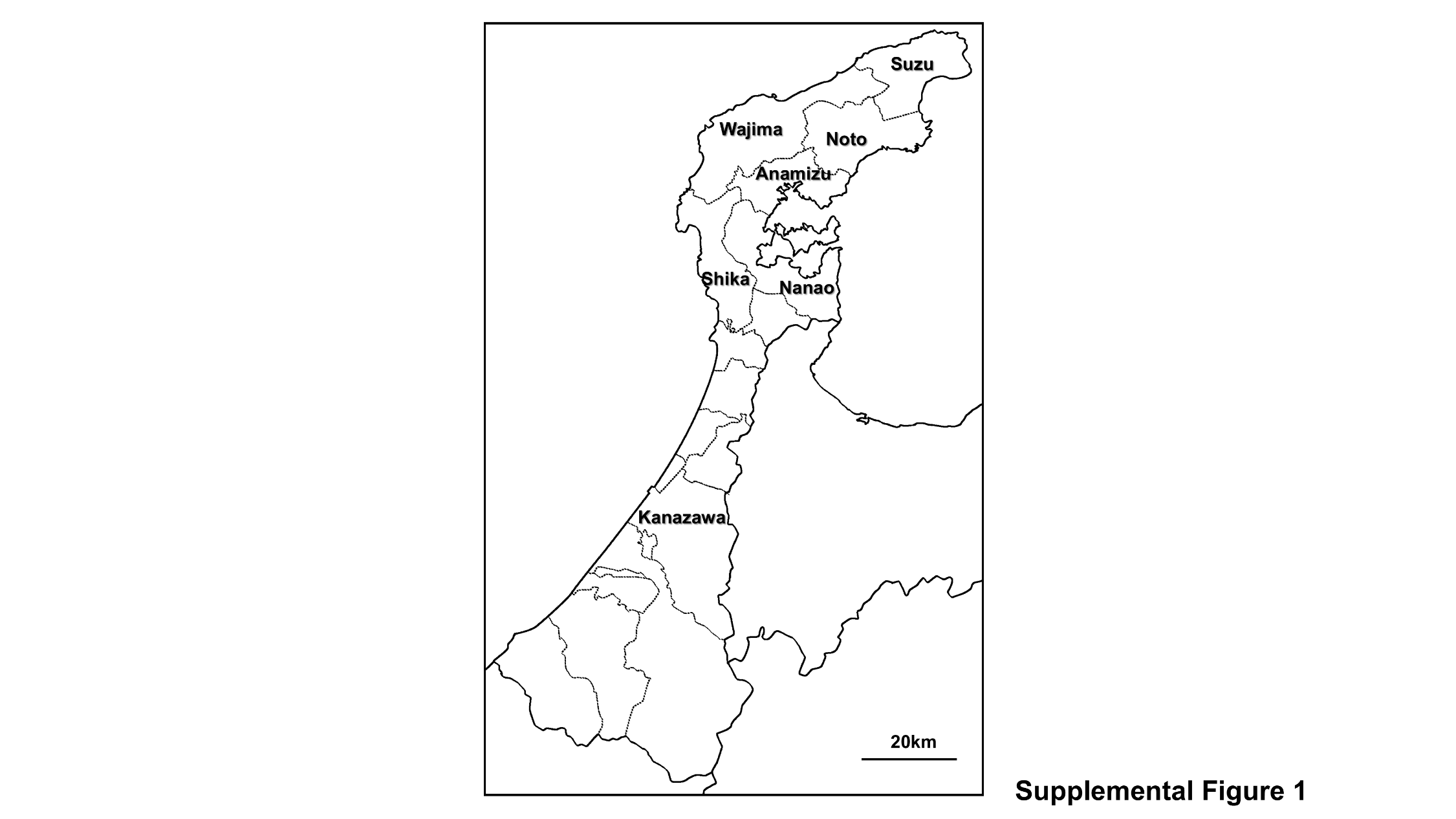


**Figure S1. Administrative map of Ishikawa Prefecture showing municipal boundaries relevant to the 2024 Noto Peninsula Earthquake.**

Highlighted municipalities include Wajima City, Suzu City, Noto Town, Nanao City, and Kanazawa City, which are mentioned in the text.

Table S1. Perinatal transport cases coordinated during the acute phase of the 2024 Noto Peninsula Earthquake

| Case | Timeline After Disaster Onset | Patient Details | Transport Mode |
| --- | --- | --- | --- |
| 1 | Day 1 | Emergency transport of a postpartum patient with septic shock. Due to a tsunami warning, helicopter flights were suspended and safety of ground routes was unconfirmed, but the patient was successfully transferred to Ishikawa Prefectural Central Hospital by the hospital’s own ambulance. | Ambulance |
| 2 | Day 1 | Transport coordination was attempted for a pregnant woman with labor onset, but transport was not possible due to a tsunami warning. She drove to her primary hospital in Nanao City by private car and delivered safely. | Liaison-Coordinated Consultation |
| 3 | Day 2 | A pregnant woman in an evacuation shelter developed painful uterine contractions. She was transported by helicopter and underwent a cesarean section due to a history of previous cesarean deliveries. | Helicopter |
| 4 | Day 2 | A 39-week pregnant woman with bleeding in an isolated area. An initial helicopter search failed to locate her. She was later found by chance by a Self-Defense Forces helicopter and transported to Wajima Hospital. The next day, she was transferred to Kanazawa by ambulance operated by the DMAT team. | Ambulance |
| 5 | Day 2 | A 37-week pregnant woman who had evacuated to an elementary school shelter was transported by helicopter. | Helicopter |
| 6 | Day 3 | A 12-week pregnant woman living in Noto experienced genital bleeding while staying overnight in her car. She was transported by helicopter and later moved to her sister’s house in Kanazawa City. | Helicopter |
| 7 | Day 3 | A 21-week pregnant woman from Osaka who was traveling and affected by the disaster in Wajima was transported by a Self-Defense Forces helicopter and later returned to her home in Osaka. | Helicopter |
| 8 | Day 3 | Two pregnant women affected by the disaster in Suzu were initially planned for helicopter transport, but were ultimately transported by ambulance to their primary general hospital in Nanao City. | Ambulance |
| 9 | Day 4 | A 35-week pregnant woman staying at the evacuation shelter developed signs of labor and was transported by a DMAT ambulance to a public general hospital in Nanao City. | Ambulance |
| 10 | Day 4 | A 29-week pregnant woman from Hakusan City, who had been living in an evacuation shelter in Wajima after the disaster, was transported by a Self-Defense Force helicopter and returned home after a medical check. | Helicopter |
| 11 | Day 4 | A postpartum woman from Kanazawa City, who had evacuated to a shelter in Wajima after the disaster, was transported by doctor helicopter due to lactation failure. | Helicopter |
| 12 | Day 5 | A 36-week pregnant woman from Wajima City was transported by doctor helicopter to Ishikawa Prefectural Central Hospital for evacuation. | Helicopter |
| 13 | Day 5 | A 23-week pregnant woman from Suzu City was transported by doctor helicopter due to bleeding. | Helicopter |
| 14 | Day 6 | A 10-week pregnant woman evacuated by private car from Suzu City to Kanazawa, received a medical check, and then moved to her parents’ home in Miyagi Prefecture. | Liaison-Coordinated Consultation |
| 15 | Day 6 | A 10-week pregnant woman with hyperemesis was transported by ambulance from Wajima to Kanazawa, hospitalized at Ishikawa Prefectural Central Hospital, and later moved to Takaoka City. | Ambulance |
| 16 | Day 7 | A 40-week pregnant woman who was at a general hospital in Nanao City was transported by ambulance to Kanazawa Medical University due to fetal distress. | Ambulance |
| 17 | Day 7 | A 35-week pregnant woman with threatened preterm labor who presented to a public general hospital in Nanao City was transferred by ambulance to Ishikawa Prefectural Central Hospital. | Ambulance |
| 18 | Day 8 | A 22-week pregnant woman with suspected preterm rupture of membranes was transported by helicopter to Ishikawa Prefectural Central Hospital | Helicopter |
| 19 | Day 10 | A pregnant woman who had undergone cervical cerclage and was staying at an evacuation shelter in Suzu traveled by private car to Kanazawa City for a scheduled prenatal check-up. | Liaison-Coordinated Consultation |
| 20 | Day 11 | A 30-week pregnant woman and her 1-year-old child, both positive for COVID-19, were transported by emergency ambulance. | Ambulance |
| 21 | Day 17 | A pregnant woman who tested positive for COVID-19 at a general hospital in Nanao City traveled by private car to Ishikawa Prefectural Central Hospital. | Liaison-Coordinated Consultation |
